# Remote Management of Atrial Fibrillation: A Case Report

**DOI:** 10.5811/cpcem.2017.4.33539

**Published:** 2017-07-14

**Authors:** Alexander Chiu, Michael Kasper, John Rimmer, Meaghan Donnelly, Yangmin Chen, Caroline Chau, Lauren Sidow, Adam Ash

**Affiliations:** Rapid Outpatient Setting Stress (ROSS) Clinical Research Organization, Saddle River, New Jersey

## Abstract

We report a case of new-onset atrial fibrillation with rapid ventricular response in a 37-year-old male who presented to the emergency department. This patient was not admitted to the hospital or placed on observation, but rather placed on a cellular outpatient 12-lead telemetry (COTLT) device with emergency response capabilities and discharged home. We define a new modality that allows these patients to be managed via telemedicine and receive care similar to that which would be rendered in a hospital or observation unit.

## INTRODUCTION

Atrial fibrillation (AF) is the most prevalent supraventricular tachycardia encountered in current hospital practice.^1^,^2^ Over the past 20 years there has been a 66% increase in hospital admissions for AF, and this growth is expected to continue due to the aging population, rising prevalence of chronic heart disease, and improvements in monitoring and diagnostic devices.^3^–^7^ By 2050, the prevalence of AF in the United States is predicted to be between 5.6 and 12 million.^8^ AF is also one of the most expensive conditions treated in U.S. hospitals today. In 2005, the national annual costs for AF treatment totaled approximately $6.65 billion.^6^ It is estimated that the current annual cost of treating one patient for AF in the U.S. is $3,600. Hospitalization is the number one expense, followed by consultations, loss of work, and paramedical procedures.^5^ Several new clinical strategies are being introduced to manage the cost of AF-related medical care, many involving the rapidly evolving field of telemedicine. We report a case of a patient with new-onset AF with rapid ventricular response (RVR) who presented to the emergency department (ED) and was managed via a cellular outpatient 12-lead telemetry (COTLT) device with emergency response capabilities in the outpatient setting, rather than being admitted to the hospital.

## CASE REPORT

A 37-year-old male with no significant past medical history presented to the ED with a chief complaint of heart palpitations. He appeared well and was hemodynamically stable. His electrocardiogram (ECG) showed AF with RVR at a rate of 129 beats per minute. Lab work, including a complete blood count and comprehensive metabolic panel were unremarkable, and his troponin was negative. He had a normal echocardiogram and received 30 mg of intravenous diltiazem over a four-hour period in the ED after which time he remained in AF with a heart rate in the 80s.

Our virtual hospital service, the Center for Remote Medical Management (CRMM), was consulted and the patient’s care was transferred to two CRMM remote physicians (an internist and a cardiologist). He was given aspirin and 150 milligrams of oral diltiazem prior to leaving the hospital. We then used a COTLT device to manage his care from home.

The device includes technology in which a 12-lead ECG heart monitor tracks real-time telemetry data sent over 3G/4G/WIFI to be monitored remotely. In the event of an emergency, the patient’s location can be pinpointed using geolocation, so that emergency services may be notified. This also allows for activation of the local catheterization lab, if necessary and available.

The patient applied the device, established a continuous connection with CRMM and was transported to his home. At home the patient had 49 episodes of AF with RVR (HR > 100 bpm), many of which were in close proximity to one another, for which the CRMM cardiologist was consulted and who directed the patient to take oral diltiazem. The image shows real-time monitoring and interpretation of one episode of home AF with RVR managed remotely by the cardiologist. All episodes of AF with RVR were rate controlled with oral diltiazem. Emergency response was never initiated. The patient was consented in writing prior to transfer of care to CRMM and is also registered with the Western Institutional Review Board.

Early the following morning while the patient was sleeping, he spontaneously converted to sinus rhythm as captured on remote telemetry. After sustained normal sinus rhythm a video cardiology consult was performed, for which non-emergent stress test and repeat echo were ordered. Given spontaneous cardioversion, lack of symptoms and lack of risk factors, the remote telemetry was discontinued and he was discharged from CRMM.

Follow-up at seven days revealed maintained sinus rhythm. The platform provided a reliable alternative to inpatient admission, with decreased cost, increased patient satisfaction, decreased exposure to nosocomial infections, and anticipated equivalent outcome of diagnostic results

## DISCUSSION

Within the rapidly emerging field of telemedicine, cardiac patients have become a major target population. There are increasing efforts to manage these patients in an outpatient setting by initiating remote cardiac management through implantable devices.^9^ As depicted in this case, the patient was first treated with intravenous diltiazem to achieve rate control and sent home with a remote monitory device.

There are no universally accepted guidelines or hospital admission criteria for patients with AF, and low-to-intermediate risk patients are often either unnecessarily admitted to the hospital, or discharged without monitoring prior to follow-up with a cardiologist. The patient in our case was low risk and likely could have been discharged home safely. However, even low-risk patients are frequently admitted to telemetry beds or placed unnecessarily in observation units. Additionally, we believe this technology to be of potential use in slightly higher risk patients, who still may not require hospital admission. We have also used it in low-risk chest pain patients.^10^

Previously, only monitoring devices have been employed in AF to reduce hospital visits and admissions. Holter monitors, for example, allow monitoring over a 24-hour to two-week period, but do not allow for remote transmission of information.^9^ Continuous-loop monitoring is also available but is a more costly approach.^9^ Subcutaneous devices have been increasing in popularity, but these are invasive and may result in procedural complications.^12^

CPC-EM Capsule

What do we already know about this clinical entity?

Although multiple devices exist for the outpatient monitoring of cardiac dysrhythmias, the majority of them are recorders that do not allow real-time analysis.

What makes this presentation of disease presentable?

We present a case of atrial fibrillation managed remotely with a real-time telemetry monitor that allowed real-time interventions.

What is the major learning point?

In the appropriate patient population, atrial fibrillation, and potentially other cardiac dysrhythmias, could be safely managed remotely via real-time telemetry monitors.

How might this improve emergency medicine practice?

Many low-to-intermediate risk patients with atrial fibrillation are unnecessarily admitted to hospitals. This strategy offers a safe alternative to admission in certain cases.

In a study by Shacham et al. in 2010, establishing long-term connections with AF patients using a remote monitoring device resulted in 80% successful AF management out of the hospital, avoiding unnecessary facility visits.^13^ Additionally, the accessibility of a call center encouraged a quicker response time in patients who were symptomatic. Larger European trials, such as the Clinical Evaluation of Remote Notification to Reduce Time to Clinical Decision (CONNECT) and Safely RedUceS RouTine Office Device Follow-Up (TRUST) trials, resulted in a significant reduction in office visits by up to 63%.^14^–^16^ Furthermore, management with remote monitoring systems had a much lower cost compared to ambulatory care and reduced the risk of stroke via earlier detection of AF.^17^ Patients felt reassured and more confident in their disease management with telemonitoring services.^18^ In REFORM, a small randomized trial, remote monitoring resulted in abundant potential to maximize healthcare resources, including reduction in hospital visits, physician time, and cost of transport to counterbalance the cost of implementing new technology.^9^

## CONCLUSION

With admissions replacement on a COTLT platform, it is possible to conduct medical operations that traditionally necessitate hospital admission (such as telemetric monitoring, serial cardiac enzymes, and other laboratory tests) outside of the facility, thus further reducing hospitalization rates and costs. These devices can provide continuous real-time communication between the patient and medical team, alert the nearest emergency medical services if necessary, and deliver the highest medical care based on the patient’s own location. With this strategy, patient care is no longer limited by the facility to which the patient is admitted and will benefit from a broader range of options, enhancing the quality of medical management. Further research on this topic is needed to determine the subset of atrial fibrillation patients who will safely benefit from this technology.

## Figures and Tables

**Image:**
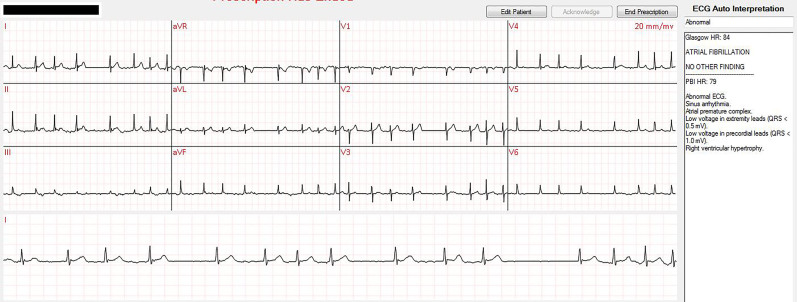
Telemetry monitoring with CardiacLinx platform exhibiting atrial fibrillation with rapid ventricular response at 135 beats per minute.

## References

[1] King DE, Dickerson LM, Sack JL (2002). Acute management of atrial fibrillation: Part I. Rate and rhythm control. Am Fam Physician.

[2] Sanoski CA, Bauman JL, DiPiro JT, Talbert RL, Yee GC, Matzke GR, Wells BG, Posey L (2014). The Arrhythmias. Pharmacotherapy: A Pathophysiologic Approach.

[3] Fuster V, Rydén LE, Cannom DS (2011). 2011 ACCF/AHA/HRS focused updates incorporated into the ACC/AHA/ESC 2006 Guidelines for the management of patients with atrial fibrillation: a report of the American College of Cardiology Foundation/American Heart Association Task Force on Practice Guidelines developed in partnership with the European Society of Cardiology and in collaboration with the European Heart Rhythm Association and the Heart Rhythm Society. J Am Coll Cardiol.

[4] Zamani P, Verdino RJ (2014). Management of Atrial Fibrillation. J Intensive Care Med.

[5] Heist EK, Mansour M, Ruskin JN (2011). Rate control in atrial fibrillation: targets, methods, resynchronization considerations. Circulation.

[6] January CT, Wann LS, Alpert JS (2014). 2014 AHA/ACC/HRS guideline for the management of patients with atrial fibrillation: executive summary: a report of the American College of Cardiology/American Heart Association Task Force on practice guidelines and the Heart Rhythm Society. Circulation.

[7] Decker WW, Smars PA, Vaidyanathan L (2008). A prospective, randomized trial of an emergency department observation unit for acute onset atrial fibrillation. Ann Emerg Med.

[8] Go AS, Mozaffarian D, Roger VL (2014). Heart disease and stroke statistics--2014 update: a report from the American Heart Association. Circulation.

[9] Varma N, Ricci RP (2013). Telemedicine and cardiac implants: what is the benefit?. Eur Heart J.

[10] Chiu A, Shumaker K, Del Corral C (2017). Remote management of low to intermediate risk chest pain: A case series. Am J Emerg Med.

[11] Gussak I, Vukajlovic D, Vuckcevic V (2012). Wireless remote monitoring of reconstructed 12-lead ECGs after ablation for atrial fibrillation using a hand-held device. J Electrocardiol.

[12] Shacham J, Birati EY, Malov N (2012). Telemedicine for diagnosing and managing paroxysmal atrial fibrillation in outpatients. The phone in the pocket. Int J Cardiol.

[13] Crossley GH, Boyle A, Vitense H (2011). The CONNECT (Clinical Evaluation of Remote Notification to Reduce Time to Clinical Decision) trial: the value of wireless remote monitoring with automatic clinician alerts. J Am Coll Cardiol.

[14] Varma N, Epstein AE, Irimpen A (2010). Efficacy and safety of automatic remote monitoring for implantable cardioverter-defibrillator follow-up: the Lumos-T Safely Reduces Routine Office Device Follow-up (TRUST) trial. Circulation.

[15] Dubner S, Auricchio A, Steinberg JS (2012). ISHNE/EHRA expert consensus on remote monitoring of cardiovascular implantable electronic devices (CIEDs). Europace.

[16] Lorenzoni G, Folino F, Soriani N (2014). Cost-effectiveness of early detection of atrial fibrillation via remote control of implanted devices. J Eval Clin Pract.

[17] Dimengo J, Stegall G (2015). Team-Based Care for External Telemonitoring in Patients with Heart Failure. Heart Fail Clin.

